# Local Difference Measures between Complex Networks for Dynamical System Model Evaluation

**DOI:** 10.1371/journal.pone.0118088

**Published:** 2015-04-09

**Authors:** Stefan Lange, Jonathan F. Donges, Jan Volkholz, Jürgen Kurths

**Affiliations:** 1 Department of Physics, Humboldt University, Berlin, Germany; 2 Potsdam Institute for Climate Impact Research, Potsdam, Germany; 3 Stockholm Resilience Center, Stockholm University, Stockholm, Sweden; 4 Institute for Complex Systems and Mathematical Biology, University of Aberdeen, Aberdeen, United Kingdom; Universiteit Gent, BELGIUM

## Abstract

A faithful modeling of real-world dynamical systems necessitates model evaluation. A recent promising methodological approach to this problem has been based on complex networks, which in turn have proven useful for the characterization of dynamical systems. In this context, we introduce three local network difference measures and demonstrate their capabilities in the field of climate modeling, where these measures facilitate a spatially explicit model evaluation. Building on a recent study by Feldhoff et al. [1] we comparatively analyze statistical and dynamical regional climate simulations of the South American monsoon system. Three types of climate networks representing different aspects of rainfall dynamics are constructed from the modeled precipitation space-time series. Specifically, we define simple graphs based on positive as well as negative rank correlations between rainfall anomaly time series at different locations, and such based on spatial synchronizations of extreme rain events. An evaluation against respective networks built from daily satellite data provided by the Tropical Rainfall Measuring Mission 3B42 V7 reveals far greater differences in model performance between network types for a fixed but arbitrary climate model than between climate models for a fixed but arbitrary network type. We identify two sources of uncertainty in this respect. Firstly, climate variability limits fidelity, particularly in the case of the extreme event network; and secondly, larger geographical link lengths render link misplacements more likely, most notably in the case of the anticorrelation network; both contributions are quantified using suitable ensembles of surrogate networks. Our model evaluation approach is applicable to any multidimensional dynamical system and especially our simple graph difference measures are highly versatile as the graphs to be compared may be constructed in whatever way required. Generalizations to directed as well as edge- and node-weighted graphs are discussed.

## Introduction

The study of complex system dynamics by means of complex network theory has thrived in recent years [1, 2]. Applications cover many branches of science as virtually any multidimensional dynamical system may be considered as a network of dynamically interacting components. For example, climate or brain functional networks are thought to consist of nodes representing climate dynamics or neural activity in different geographical or brain regions and links between them characterizing interregional covariabilities [3–7].

Recently, Feldhoff et al. [8] have proposed to use complex networks for climate model evaluation comparing functional networks built from simulated and observed climate data. In contrast to most commonplace methods, this approach offers a multivariate evaluation perspective and the authors have demonstrated its complementarity to traditional univariate methods based on mean values and variances. Beyond climate science, the fundamental idea of a model evaluation based on functional networks applies to numerous kinds of real-world dynamical systems. For instance, it could be used to evaluate models of social dynamics [9], financial markets [10], neural activity [11, 12] or genetic regulatory systems [13, 14].

A quantification of modeling accuracy following the new paradigm requires measures of difference between complex networks with known node correspondence. Those put forward thus far usually compare community [15, 16] or neighborhood [8, 17–19] structures and boil down network dissimilarities to one single number. While this is advantageous from a model rating point of view it is of limited use when an evaluation aims at model improvement. In that latter context a more detailed account of network differences is required in order to identify those system components whose interaction with the rest of the system is simulated most deficiently. This motivates the work on local network difference measures presented in this article.

There already have been methods proposed in the literature to quantify the structural similarity of network nodes. The older and simpler concept of structural equivalence considers nodes similar if they have many common network neighbors [20, 21] while the more recent concept of regular equivalence considers nodes similar if the nodes they are connected to are themselves similar [22, 23]. The latter concept requires transitivity of similarity through network links and therefore does not apply to networks where links represent dissimilarity. Since such networks can occur in the model evaluation context as exemplified in the application section we define local network difference measures in accordance with the concept of structural equivalence. To our knowledge, we are the first to employ this concept to quantify the structural (dis)similarity of nodes from different networks.

To illustrate the general scope of application of the new evaluation approach, let us consider an *N*-dimensional dynamical system of which we know the time evolution within a certain time frame, i.e. we know *x*_*i*_(*t*) for *i* = 1, …, *N* and *t* = 1, …, *M*. A description of the system dynamics according to the complex network approach is then based on statistical relationships between the time series as quantified by some measure of statistical dependence. Measures that have been used for this purpose include linear correlations [24, 25] as well as nonlinear measures of synchronization [26, 27] and mutual information [4, 28]. The dependence measure of choice is calculated for every pair of time series, which yields an *N* × *N* matrix of relationship coefficients *r*_*ij*_.

Translated into network language, the *N* different components or dimensions of the system are the nodes or vertices of a network and the coefficients *r*_*ij*_ constitute links or edges of varying strength between them. This identification enables the application of network structure analysis tools, aiming at an improved understanding of the collective dynamics of the system under study. To simplify the analysis, the matrix of relationship coefficients is frequently mapped to a binary adjacency matrix with components *a*_*ij*_ = 1 if nodes *i* and *j* are connected and *a*_*ij*_ = 0 otherwise [29, 30]. These unweighted links are typically assigned according to the significance of the respective statistical relationship.

Now let us consider the system to be a real-world one that is to be modeled, i.e. let us assume we have $x_{i}^{A}(t)$ from measurements and $x_{i}^{B}(t)$ from model simulations and that we want to assess how close the simulated dynamics are to the observed. Then the complex network approach allows for an evaluation of the interdimensional covariabilities of the system by comparing $\left(r_{ij}^{A}\right)$ with $\left(r_{ij}^{B}\right)$ or $\left(a_{ij}^{A}\right)$ with $\left(a_{ij}^{B}\right)$.

In the following section we define local difference measures between complex networks represented by matrices of the (*r*_*ij*_) and (*a*_*ij*_) type. We then demonstrate the capabilities of these measures in a climate model evaluation case study. Potential generalizations of our theory and applications beyond climate model evaluation are discussed at the end.

## Local network difference measures

In this section, after briefly recalling some basic graph theoretical notions, we first define a local version of the Hamming distance [29, 31] between binary adjacency matrices, describe a practical problem coming along with its application, and present a solution, which culminates in the definition of a new local network difference measure. Subsequently, we discuss the cases of edge-weighted and node-weighted networks. For the sake of simplicity, only undirected networks are considered throughout this section; a generalization to directed graphs is mentioned in the discussion section.

We use the superscripts *A* and *B* to discriminate between the networks to be compared. According to the model evaluation context we assume that these networks share a common set of *N* nodes, and that they have been constructed from (*A*) observed and (*B*) simulated data following the same recipe, as outlined in the introduction and exemplified in the application section. Because the diagonal elements of the (*r*_*ij*_) and (*a*_*ij*_) matrices encode statistical relationships between identical time series, they are disregarded by all difference measures defined hereafter.

### Simple graphs

Based on the above, let us consider two networks represented by symmetric binary adjacency matrices $\left(a_{ij}^{A}\right)$ and $\left(a_{ij}^{B}\right)$ with diagonals set to zero. Such networks are known as simple graphs in the mathematical literature. Nodes which are connected are called neighbors. The set of all nodes connected to node *i* is *i*’s neighborhood. The number of its neighbors is *i*’s degree and can be written as

$$\begin{array}{c} k_{i}=\sum_{j=1}^{N}a_{ij}. \end{array}$$ (1)

The average degree over all nodes, divided by *N* − 1, is called the link density *ρ* of the network.

The *global Hamming distance* (GHD) between two simple graphs *A* and *B* has been defined to be [29, 31]

$$\begin{array}{c} H\left(A,B\right)=\sum_{i,j=1}^{N}a_{ij}^{A}\;\mathrm{XOR}\;a_{ij}^{B}, \end{array}$$ (2)

$$\begin{array}{c} a_{ij}^{A}\;\mathrm{XOR}\;a_{ij}^{B}={\left(a_{ij}^{A}-a_{ij}^{B}\right)}^{2}=a_{ij}^{A}+a_{ij}^{B}-2\;a_{ij}^{A}a_{ij}^{B}. \end{array}$$ (3)

We define its local version by

$$\begin{array}{c} H_{i}\left(A,B\right)=\sum_{j=1}^{N}a_{ij}^{A}\;\mathrm{XOR}\;a_{ij}^{B}, \end{array}$$ (4)

relating the neighborhoods of node *i* in both networks. The *local Hamming distance* (LHD) *H*_*i*_(*A*, *B*) counts the number of nodes, which are either a neighbor of *i* in network *A* but not in *B* or vice versa. Global and local Hamming distances are related by *H*(*A*, *B*) = ∑_*i*_ *H*_*i*_(*A*, *B*). Differently from [29], we refrain from normalizing the LHD to the number of nodes in this section, because it would make the following mathematics less convenient.

Fig. 9(j) shows LHDs between climate networks over South America with degree fields displayed in Figs. 4(i) and 4(j). (We are going to elaborate on the background of these networks in the application section; for the moment the reader is asked to take them as given.) We observe large LHDs in locations with large degree discrepancies, for example around 10°S, 50°W. Moreover, larger LHDs tend to occur in locations with larger degrees. For instance, small degrees coincide with small LHDs along the western coast of South America.

These observations indicate that the LHD is not an ideal local network difference measure. Imagine two nodes *i*, *j* with $k_{i}^{A}=k_{i}^{B}\neq k_{j}^{A}=k_{j}^{B}$. Equal LHD values at these nodes would mean different relative agreements of neighborhoods. It is therefore difficult to interpret LHD values without considering the degrees. We would like to have a more intuitive difference measure which quantifies the dissimilarity of neighborhoods relative to their size. An equivalent problem has been encountered by those studying vertex similarity concepts [21]. While, to our knowledge, this community has always made do with ad hoc renormalizations [32–34], we take a different approach in the following.

Our point of departure is a statistical null model which explains the observed LHD-degree dependence: Let *i* be some fixed node with degrees $k_{i}^{A}$ in graph *A* and $k_{i}^{B}$ in graph *B*, and let us assume *i*’s neighborhoods in *A* and *B* to be statistically unrelated. This can be modeled considering $a_{ij}^{A}$ and $a_{ij}^{B}$ (*j* ≠ *i*) to be random binary variables that are statistically independent between networks and equal to one with identical probability within networks. Using Eq. (3), this null model yields an LHD expectation value of $\left\langle H_{i}(A,B)\right\rangle =k_{i}^{A}+k_{i}^{B}-2\;k_{i}^{A}k_{i}^{B}/(N-1)$.

Beyond illustrating the relationship between LHD and degree, this null model can be used to define a new local network difference measure, which is degree-independent. To that end, we relate the actually measured LHD value *H*_*i*_(*A*, *B*) to the null model probability distribution of possible LHD values for the degrees $k_{i}^{A}$ and $k_{i}^{B}$. More specifically, *H*_*i*_(*A*, *B*) is mapped to its null model *p*-value, i.e. to the probability of the LHD to take a value less than or equal to *H*_*i*_(*A*, *B*), if *i*’s neighborhoods of size $k_{i}^{A}$ in *A* and $k_{i}^{B}$ in *B* were statistically unrelated.

We now derive a formula for those *p*-values, i.e. we derive the cumulative distribution function (CDF) of LHDs generated by our null model. According to Eq. (3) we can write

$$\begin{array}{c} H_{i}\left(A,B\right)=k_{i}^{A}+k_{i}^{B}-2\sum_{j\neq i}a_{ij}^{A}a_{ij}^{B}. \end{array}$$ (5)

The sum in Eq. (5) counts the number of common neighbors of *i* in *A* and *B*. We denote this number by *N*_*i*_(*A*, *B*) and have

$$\begin{array}{c} N_{i}\left(A,B\right)=\frac{1}{2}\left(k_{i}^{A}+k_{i}^{B}-H_{i}\left(A,B\right)\right). \end{array}$$ (6)

For fixed $k_{i}^{A}$ and $k_{i}^{B}$, this implies a one-to-one correspondence between *N*_*i*_(*A*, *B*) and *H*_*i*_(*A*, *B*), with changes of the former by +1 corresponding to changes of the latter by −2. Let us denote the null model generated LHDs by $h=k_{i}^{A}+k_{i}^{B}-2n$ in the spirit of Eq. (6). The null model probability of node *i* to have exactly *n* common neighbors in the two networks is equal to the probability of having exactly *n* successes in $k_{i}^{B}$ draws without replacement from a population of size *N* − 1 containing $k_{i}^{A}$ successes. This probability is given by

$$\begin{array}{c} P\left(n;k_{i}^{A},k_{i}^{B},N\right)=\left(\begin{array}{c} k_{i}^{A}\\ n \end{array}\right)\left(\begin{array}{c} N-1-k_{i}^{A}\\ k_{i}^{B}-n \end{array}\right){\left(\begin{array}{c} N-1\\ k_{i}^{B} \end{array}\right)}^{-1}, \end{array}$$ (7)

the probability density function (PDF) of a hypergeometric distribution [35, 36]. Note that $P\left(n;k_{i}^{A},k_{i}^{B},N\right)$ is symmetric with respect to exchanging $k_{i}^{A}$ with $k_{i}^{B}$.

The desired *p*-value formula results from Eq. (7) via

$$\begin{array}{cc} & p\left(H_{i}\left(A,B\right);k_{i}^{A},k_{i}^{B},N\right)\\ & \;=P\left(h\leq H_{i}\left(A,B\right);k_{i}^{A},k_{i}^{B},N\right)\\ & \;=P\left(n\geq N_{i}\left(A,B\right);k_{i}^{A},k_{i}^{B},N\right)\\ & \;=\sum_{n=N_{i}\left(A,B\right)}^{\min\left\{k_{i}^{A},k_{i}^{B}\right\}}P\left(n;k_{i}^{A},k_{i}^{B},N\right). \end{array}$$ (8)

**Fig 1:**
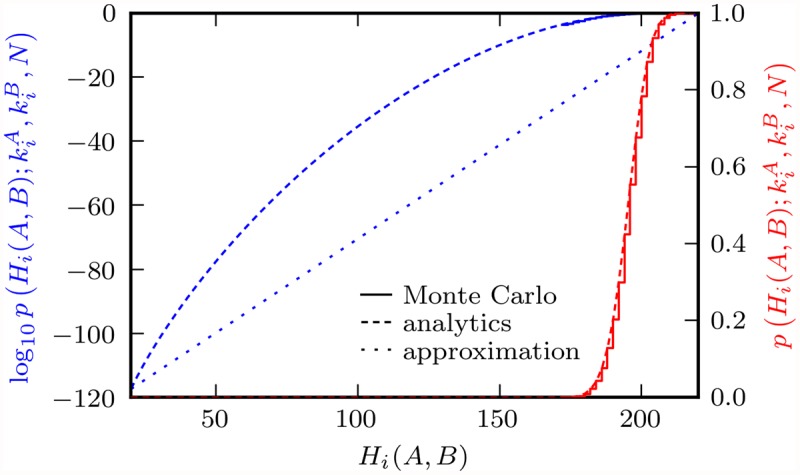
Null model *p*-values of *H_i_*(*A, B*) for $k_{i}^{A}$ = 120, $k_{i}^{B}$ = 100, *N* = 1000, both from 10000 Monte Carlo trials (solid lines) and from Eq. (8) (dashed lines). The dotted line is the log *p* approximation given in Eq. (16).

In order to numerically evaluate this complementary CDF of a hypergeometric distribution, we use the HyperQuick algorithm [37]. This algorithm has the advantage of being easily logarithmized, which is important because of the following observation. The *p*-values of LHDs between climate networks on observational and modeled rainfall data studied in the application section turn out to typically be many orders of magnitude smaller than one. In other words, the climate models studied here are much better than our null model. Since the latter corresponds to the Configuration Random Network Model [1], this confirms the GHD findings by Feldhoff et al. [8]. To still be able to visualize *p*-values, we move to their logarithms. The agreement of our analytical result for $p\left(H_{i}(A,B);k_{i}^{A},k_{i}^{B},N\right)$ and its Monte Carlo simulation is depicted in Fig. 1.

Unfortunately, log *p* is degree-dependent, again. For instance, for its minimum value

$$\begin{array}{cc} & \min_{h}\log\;p\left(h;k_{i}^{A},k_{i}^{B},N\right)\\ & \;=\log\;P\left(\min\left\{k_{i}^{A},k_{i}^{B}\right\};k_{i}^{A},k_{i}^{B},N\right)\\ & \;=\sum_{i=0}^{\min\left\{k_{i}^{A},k_{i}^{B}\right\}-1}\log\frac{\max\left\{k_{i}^{A},k_{i}^{B}\right\}-i}{N-1-i} \end{array}$$ (9)

we find that, per simultaneous increment of both degrees, min_*h*_ log *p* decreases by approximately −log *ρ* for small link densities *ρ*. To overcome this problem, we rescale log *p* by its minimum value, thus defining a new local network difference measure

$$\begin{array}{c} D_{i}\left(A,B\right)=1-\frac{\log\;p(H_{i}\left(A,B\right);k_{i}^{A},k_{i}^{B},N)}{{\text{min}}_{h}\;\text{log}\;p\left(h;k_{i}^{A},k_{i}^{B},N\right)}, \end{array}$$ (10)

which we call the *degree-conditional neighborhood dissimilarity* (DND). Note that *D*_*i*_(*A*, *B*) is only defined if $k_{i}^{A}$ and $k_{i}^{B}$ are both positive. The definition is independent of the base of the logarithm.

The DND can only take values in [0, 1], with a value of zero (one) meaning maximally (minimally) overlapping neighborhoods of node *i* in the two networks, given the degrees. Note that *D*_*i*_(*A*, *B*) = 0 does not imply a local agreement between networks. Rather, it means the greatest possible agreement for given degree differences. The DND should therefore always be considered together with either the LHD or the degrees of the compared networks.

In the case of a comparison of spatially embedded networks, the LHD shows embedding artifacts such as boundary effects [38], which are inherited from the degree. Since the transformation of LHDs to DNDs frees the former of its degree dependence, the latter does not show the according artifacts. As an example, compare the LHDs between regional climate networks in Fig. 9(i-l) with their respective DNDs in Fig. 9(m-p).

### Edge-weighted graphs

As outlined in the introduction, simple graphs describing complex system dynamics usually result from a binarization of a matrix of statistical relationship coefficients *r*_*ij*_. Such a procedure artificially degrades the network information content. Moreover, it usually involves the introduction of binarization parameters, which many properties of the resulting simple graphs depend on.

In the model evaluation context it may be desirable to omit this problematic procedure, i.e. to directly evaluate the simulation accuracy with respect to the (*r*_*ij*_) matrix. To that end a local network difference measure of such matrices is needed. We will now introduce one for *r*_*ij*_ being Pearson correlation coefficients.

In formal analogy to the (normalized) local Hamming distance, we define the *local correlation distance* (LCD) by

$$\begin{array}{c} C_{i}\left(A,B\right)=\sqrt{\frac{1}{N-1}\sum_{j\neq i}{\left(F\left(r_{ij}^{A}\right)-F\left(r_{ij}^{B}\right)\right)}^{2}}, \end{array}$$ (11)

$$\begin{array}{c} F:(-1,1)\to (-\infty ,\infty ),\;r\mapsto \mathrm{arctanh}\;r. \end{array}$$ (12)

The use of the Fisher transformation [39] *F* in the LCD definition is motivated as follows. The confidence interval width of Pearson correlation estimates depends on their value. An *r*_*ij*_ value around 0 usually has a wider confidence interval than a value close to ±1. Therefore, at correlation coefficient values around 0, we expect $|r_{ij}^{A}-r_{ij}^{B}|$ to be greater than at values close to ±1. The use of Fisher transformed coefficients balances this disparity. For normally distributed time series, the standard estimation error of *F*(*r*_*ij*_) is approximately independent of *r*_*ij*_ [39–42]. Thus, in the definition of Eq. (11), differences of *r*_*ij*_ values close to ±1 contribute as much to *C*_*i*_(*A*, *B*) as those at values around 0.

Furthermore, using the non-transformed correlation coefficients in Eq. (11) would make *C*_*i*_(*A*, *B*) dependent on $\sum_{j\neq i}r_{ij}^{X}$, *X* = *A*, *B*, analogously to the LHD-degree dependence on simple graphs. Employing the Fisher transformation prevents this dependence and renders a DND analog for edge-weighted graphs unnecessary.

For later reference, we define the *global correlation distance* (GCD) by *C*(*A*, *B*) = ∑_*i*_ *C*_*i*_(*A*, *B*)/*N*.

Basically, the ansatz just outlined for the Pearson correlation may be applied to any dependence measure. However, as we have tried to argue, prior to any distance calculation, dependence coefficients should be transformed such that their uncertainties become value-independent. This should be seen as an incentive to develop currently lacking estimation error theories for statistical dependence measures.

### Node-weighted graphs

To conclude this section, we shortly discuss a generalization of our approach to networks featuring node weights *w*_*i*_, which we assume to be equal in networks *A* and *B*. For instance, nodes of a climate network may represent differently sized portions of the earth’s surface.

Given such networks, it is desirable to define network measures, that take node weights into account. Following a general solution to this problem proposed in [43], we obtain the node-weighted version $H_{i}^{*}$ of the LHD,

$$\begin{array}{c} H_{i}^{*}\left(A,B\right)=\sum_{j=1}^{N}w_{j}\left(a_{ij}^{A}\;\mathrm{XOR}\;a_{ij}^{B}\right). \end{array}$$ (13)

Averaging $H^{*}(A,B)=\sum_{i}w_{i}H_{i}^{*}(A,B)$ yields the node-weighted version of the GHD [8]. Analogously, we can define a node-weighted LCD by

$$\begin{array}{c} C_{i}^{*}\left(A,B\right)=\sqrt{\frac{1}{W_{i}}\sum_{j\neq i}w_{j}{\left(F\left(r_{ij}^{A}\right)-F\left(r_{ij}^{B}\right)\right)}^{2}} \end{array}$$ (14)

with *W*_*i*_ = ∑_*j* ≠ *i*_ *w*_*j*_.

Deriving a node-weighted version of the DND is less straightforward. In formal analogy to Eq. (10), $D_{i}^{*}(A,B)$ should be a function of $H_{i}^{*}(A,B)$ and the node-weighted degrees $k_{i}^{A*},k_{i}^{B*}$ with [43]

$$\begin{array}{c} k_{i}^{*}=w_{i}+\sum_{j\neq i}w_{j}a_{ij}. \end{array}$$ (15)

In contrast to *k*_*i*_ though, any rearrangement of *i*’s neighborhood potentially changes $k_{i}^{*}$, depending on the spectrum of node weights. The combinatoric null model approach that led from LHD to DND is thus unsuitable for a derivation of $D_{i}^{*}(A,B)$. We could numerically compute the node-weighted null model CDF using Monte Carlo techniques, but a general closed-form analytical solution appears to be out of reach.

As the node-weighted DND should equal the DND in the case of constant node weights, we can nevertheless provide an approximation to the unknown $D_{i}^{*}(A,B)$ based on an approximation to *D*_*i*_(*A*, *B*): In Fig. 1 we observe that the function $\log\;p\left(h;k_{i}^{A},k_{i}^{B},N\right)$ may be approximated by a straight line through its extreme points, preserving its strict monotonicity and range of values. Substituting log *p* in Eq. (10) accordingly yields

$$\begin{array}{c} D_{i}\left(A,B\right)\approx \frac{H_{i}\left(A,B\right)-\min\;h}{\max\;h-\min\;h} \end{array}$$ (16)

with minimum and maximum LHD values for fixed degrees of $\min\;h=|k_{i}^{A}-k_{i}^{B}|$ and $\max\;h=k_{i}^{A}+k_{i}^{B}$, respectively, thus

$$\begin{array}{c} D_{i}\left(A,B\right)\approx \frac{H_{i}\left(A,B\right)-\left|k_{i}^{A}-k_{i}^{B}\right|}{2\min\{k_{i}^{A},k_{i}^{B}\}} \end{array}$$ (17)

$$\begin{array}{cc} & =1-\frac{N_{i}\left(A,B\right)}{\min\{k_{i}^{A},k_{i}^{B}\}}. \end{array}$$ (18)

Since the hypergeometric distribution is log-concave [44], $\log\;p\left(h;k_{i}^{A},k_{i}^{B},N\right)$ is a concave function in *h* [45]. Consequently, the approximate DND values are always less than or equal to their true counterparts. Expression (18) shows that the DND is approximately equivalent to the structural vertex similarity measure introduced in [34].

For fixed node-weighted degrees, the minimum and maximum node-weighted LHDs are $\min\;h^{*}=|k_{i}^{A*}-k_{i}^{B*}|$ and $\max\;h^{*}=k_{i}^{A*}+k_{i}^{B*}-2w_{i}$, respectively. Hence, an approximation to the node-weighted DND is given by

$$\begin{array}{c} D_{i}^{*}\left(A,B\right)\approx \frac{H_{i}^{*}\left(A,B\right)-\left|k_{i}^{A*}-k_{i}^{B*}\right|}{2(\min\left\{k_{i}^{A*},k_{i}^{B*}\right\}-w_{i})}. \end{array}$$ (19)

## Application to climate model evaluation

As alluded to in the introduction, Feldhoff et al. [8] have demonstrated the complex networks approach to climate model evaluation, comparing the performance of two regional climate models (RCMs) over South America. Specifically, the authors constructed networks of 2m temperature, precipitation, 500hPa geopotential height and sea level pressure time series from hindcast climate simulations and ERA-Interim reanalysis data [46]. The GHD between model and reanalysis networks was employed as the error measure, with respect to which the statistical model STARS outperformed the dynamical model CCLM for all variables except the 500hPa geopotential height.

The ERA-Interim reanalysis data are the result of a 12-hourly assimilation of historical meteorological observations and their integration by a numerical weather prediction (NWP) model. The data quality varies from variable to variable and depends on the relative influences of the observations and the NWP model [47–49]. Among the variables considered by Feldhoff et al. [8], precipitation is the most uncertain one as it is difficult to model and since no precipitation measurements are included in the assimilation of ERA-Interim.

Therefore, in the following, we reevaluate the quality of networks of precipitation time series modeled by CCLM and STARS, this time with respect to networks of observed precipitation data, and using our new local network difference measures. Before discussing results, we briefly introduce the models and the observational dataset, specify the precipitation climatology to be modeled, and describe our network construction methodology.

### Models

The RCMs CCLM [50, 51] and STARS [52, 53] differ fundamentally in their approach to climate modeling. While the statistical model STARS takes climate data as input and stochastically resamples them such that a prescribed trend of some variable is matched, the dynamical model CCLM is the climate version of a NWP model, i.e. it numerically solves the differential equations governing the dynamics of the atmosphere under prescribed external drivers such as the solar radiative forcing and greenhouse gas concentrations.

For detailed descriptions of both models and the experimental setup of the evaluation see [8] and references therein. In short, ERA-Interim reanalysis data was used to force both models. With STARS, daily mean value time series from 1979 through 1995 were resampled in order to reproduce the 1996–2011 temperature trends. CCLM was run on the CORDEX South America domain [54, 55], simulating 1979 through 2011 to ensure a proper model spin-up during the first half of the simulation period. The daily mean precipitation time series were then conservatively interpolated [56] back to the ERA-Interim grid in order to enable network constructions on a common node set. We also include the ERA-Interim reanalysis data (ERAI) in our reevaluation.

While there is only one CCLM run and one reanalysis dataset, with STARS an ensemble of 200 climate realizations was generated. All quantities evaluated in the following are calculated for each realization individually and then averaged across the ensemble, i.e. any STARS result shown represents the respective ensemble average.

### Precipitation data and climatology

Arguably the best observational precipitation dataset available for tropical South America is the Tropical Rainfall Measuring Mission (TRMM) 3B42 V7 daily satellite product, which starts in 1998 and comes at a native resolution of 0.25° [57]. The product is based on measurements by radar, infrared and microwave sensors aboard numerous satellites, and calibrated by station data. While it is available up to 50° latitude, the TRMM precipitation radar data only extend to 36° latitude so that we constrain our analysis to latitudes north of 40°S, where the data are most reliable. To facilitate a proper network comparison, the TRMM data are conservatively remapped to the native ERA-Interim grid which has a resolution of about 0.7°.

**Fig 2:**
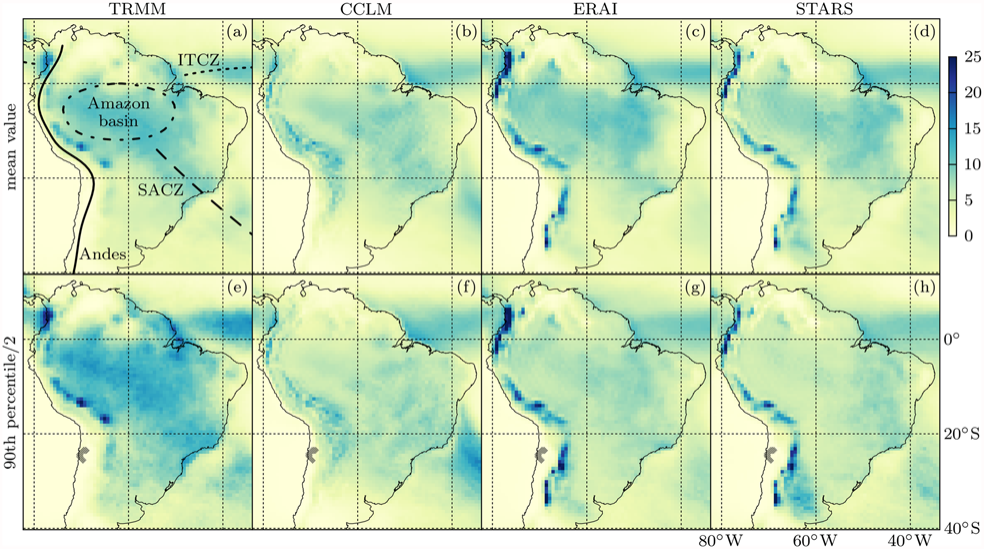
DJF 1998–2011 mean values (top) and 90th percentiles (bottom) of daily precipitation amounts in mm/day as measured by TRMM and modeled by CCLM, ERA and STARS (from left to right). Note that the 90th percentiles have been divided by 2 to fit into the same scale as the mean values. Grid cells with 90th percentiles equal to zero in any of the observed or simulated datasets are hatched in (e-h). The locations of the Andes (solid), the Amazon basin (dash-dotted), the ITCZ (dotted) and the SACZ (dashed) are sketched in (a).

We concentrate our analysis on the austral summer season DJF, when a monsoon system develops over tropical South America and provides for most of the annual precipitation [58–60]. The region under study tightly encompasses the South American mainland and is depicted in Fig. 2, where we show the DJF mean values and 90th percentiles of daily precipitation amounts as measured by TRMM and modeled by CCLM, ERAI and STARS from 1998 through 2011. The TRMM data exhibit the typical pattern of abundant rainfall in the Intertropical and the South Atlantic Convergence Zone (ITCZ and SACZ, respectively), and along the eastern slopes of the Andes.

These main rainfall patterns are replicated by the models but we find substantial differences in intensities. Regarding the seasonal mean values, CCLM mostly underestimates rainfall, while ERAI and STARS are closer to TRMM except along the Andes, where we find strong overestimations. The 90th percentiles, which quantify the intensity of extreme rain events, are mostly and substantially underestimated by all models. While TRMM shows values greater than twice the respective mean values throughout the study region, the models simulate a smaller ratio at most locations. Such intensity underestimations of extreme rainfall events are shared by many climate models [61].

### Network construction

Climate networks are constructed from space-time series of daily precipitation amounts during the 1998–2011 austral summer seasons. Since we discard the incomplete seasons January-February 1998 and December 2011 from our analysis, this implies time series lengths of *M* = 1170 days. Our domain/network comprises *N* = 5460 grid cells/nodes. We focus on networks without node weights to enable an application of the DND. This is reasonable since LHD and GHD results do not change qualitatively when node weights representing grid cell sizes are introduced, which is because grid cells do not vary much in size within the domain. To capture different aspects of the precipitation dynamics, we apply two statistical dependence measures—the Event Synchronization (ES) [62] and the Spearman Rank correlation coefficient (SR) [63].

**Fig 3:**
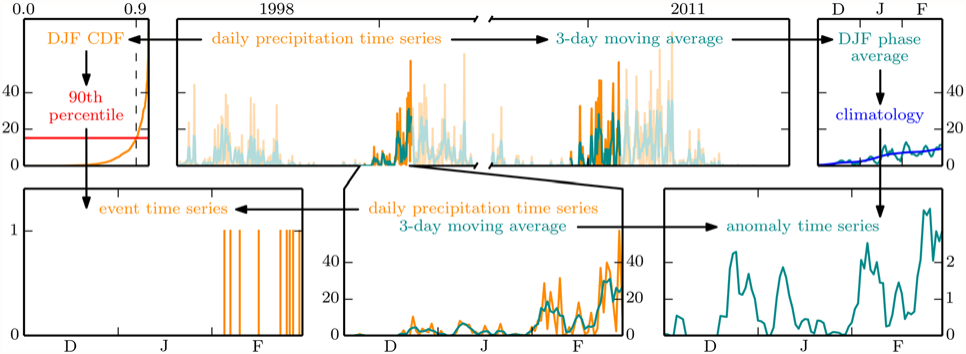
Schematic of local precipitation time series preprocessing to yield extreme event and anomaly time series. We start with daily precipitation values from the austral summer seasons DJF of 1998 through 2011 (dark orange, top middle) and their 3-day moving averages (dark cyan, top middle). The 90th percentile of all of those daily values (red, top left) is the threshold used to define extreme events (bottom left). The phase-averaged 3-day moving averages, further smoothed by a 7-day Gaussian filter (blue, top right) serve as a climatological DJF time series, to which we scale the 3-day moving averages of each individual season to obtain anomalies (bottom right).

The ES may be used to analyze the spatial synchronicity structure of extreme precipitation events [27] which has been done over South America based on the TRMM data used in this study [64]. We adopt the network construction methodology described in the latter study. It is based on a transformation of precipitation time series to binary extreme event time series as depicted in Fig. 3. At each location, daily precipitation above the 90th percentile of its climatological (DJF 1998–2011) distribution is defined as an extreme event. Grid cells at which the 90th percentile is zero in any of the observed or simulated datasets [hatched in Fig. 2(e-h)] are discarded from the analysis. Between two extreme event time series at different locations, the ES then quantifies the degree of event synchronization, with two events contributing to ES if they can be uniquely associated within a maximum period of 3days. Since no variance-stabilizing transformation is known for ES, we confine the ES network evaluation to the respective simple graph whose links represent the *ρ* = 2% highest ES values and which we call ESp.

Besides focusing on extremes, we also aim at evaluating the general spatiotemporal precipitation dynamics. To that end we employ the SR approach proposed by Feldhoff et al. [8], which requires preprocessing of the original precipitation values to anomalies with respect to the 1998–2011 climatology (Fig. 3). We first apply a 3-day moving average filter to the daily precipitation time series so as to make them represent synoptic weather situations. In order to prevent trivial correlations due to seasonality, these smoothed time series are then rendered approximately stationary in mean and variance. We achieve this by rescaling the time series of each individual DJF season to their respective climatological (1998–2011) average smoothed by a 7-day Gaussian filter (blue curve in Fig. 3). We prefer this division by climatological daily values over their more conventional subtraction, because only the scaling guarantees a common rank of all zero precipitation events. To avoid division by zero we define a minimum climatological value of 0.1mm/day, which customarily is referred to as the smallest measurable daily precipitation amount. Statistical dependences between the resulting anomaly time series are then quantified by the SR at lag zero; ties are handled according to the mid-rank method [65]. From the matrix of rank correlation coefficients we derive three networks. The matrix as it stands defines an edge-weighted network, which we denote by SR. Simple graphs representing the 2% most positive and the 2% most negative SR values are defined to disentangle these two different kinds of correlation, and are denoted by SRp and SRn, respectively.

Altogether and schematically, our networks are constructed as follows:

$$\begin{array}{c} \left(x_{i}\left(t\right)\right)\overset{P}{\mapsto }\left(y_{i}\left(t\right)\right)\overset{S}{\mapsto }\left(r_{ij}\right)\overset{T}{\mapsto }\left(a_{ij}\right), \end{array}$$ (20)

where *x*_*i*_(*t*) are the local daily precipitation time series, *P* represents their preprocessing to anomaly or extreme event time series *y*_*i*_(*t*) (cf. Fig. 3), *S* = SR, ES marks the application of a statistical dependence measure to all pairs of time series which results in a matrix (*r*_*ij*_) of correlation or synchronization strengths, and *T* denotes the thresholding that yields the simple graph adjacency matrices (*a*_*ij*_) according to

$$\begin{array}{c} a_{ij}=\left\{\begin{array}{cc} \Theta [\tau \left(\rho \right)-r_{ij}] & \text{for}\;\text{SRn,}\\ \Theta \left[r_{ij}-\tau \left(\rho \right)\right]-\delta_{ij} & \text{for}\;\text{SRp,}\;\text{ESp,} \end{array}\right. \end{array}$$ (21)

with the Heaviside function Θ, the Kronecker delta *δ*_*ij*_, and the threshold *τ* set such as to obtain the desired link density *ρ*.

It should be noted that the modeling accuracy of these networks is, in principle, independent of general rain amount biases. Two pairs of time series can have the same correlations even if standard deviations or means of the time series differ. Similarly, two pairs of (extreme) event time series can agree in synchronicity despite disagreement in the event defining threshold. Therefore, the discrepancies between observed and modeled precipitation mean values and 90th percentiles we see in Fig. 2 do not preclude agreement in the SR or ES network structures.

### Network topologies

**Fig 4:**
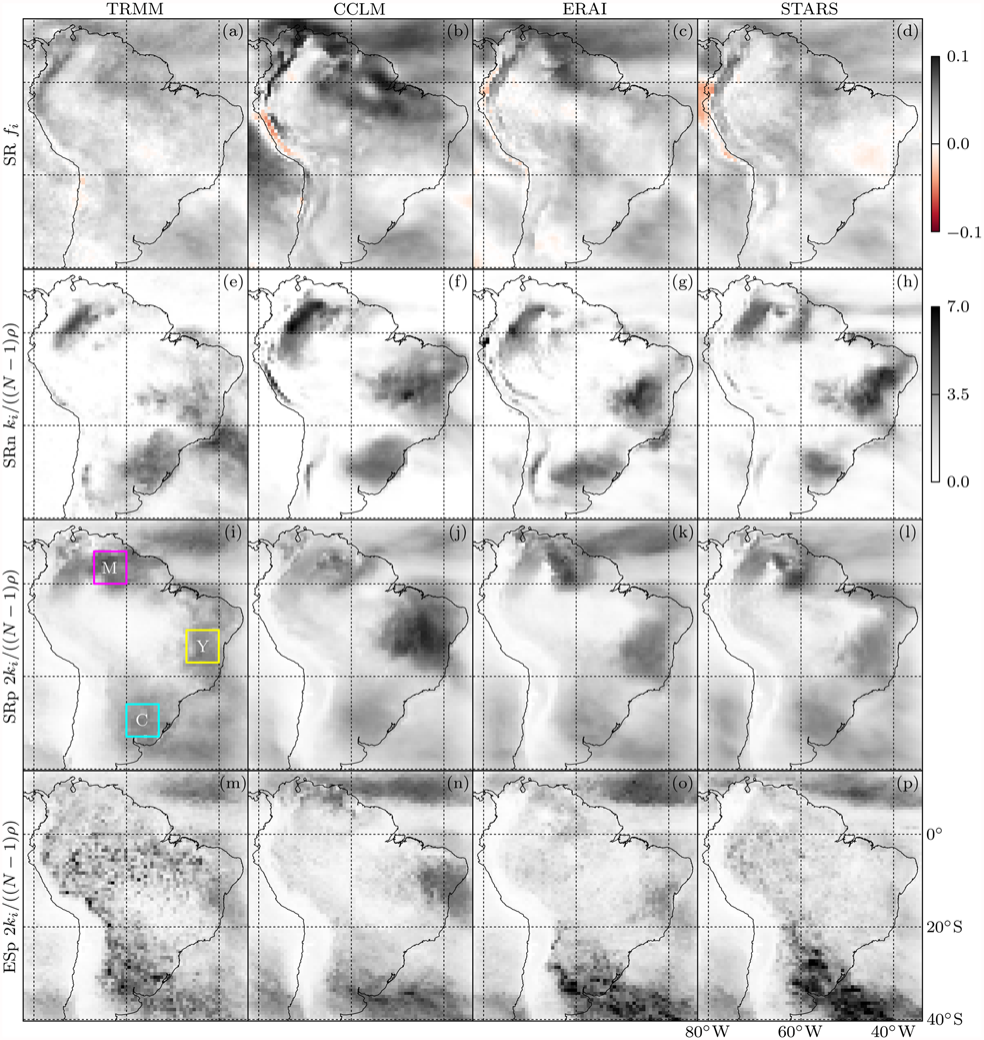
Mean Fisher transformed SRs [top, Eq. (22)] and degrees [Eq. (1)] of SRn, SRp and ESp simple graphs (from top to bottom) from rainfall data measured by TRMM and modeled by CCLM, ERA and STARS (from left to right). Degrees are shaded according to the lower color scale and have been rescaled to their average (*N*−1)*ρ*; note further that the ESp and SRp degrees have been multiplied by 2 to fit them into one scale with the SRn degrees. The three colored 7° × 7° boxes in (i) define the regions referred to in the text and in Fig. 6.

Prior to an application of our novel network difference measures we investigate the individual network topologies starting with mean Fisher transformed rank correlations,

$$\begin{array}{c} f_{i}=\frac{1}{N-1}\sum_{j\neq i}F\left(r_{ij}\right), \end{array}$$ (22)

and simple graph degrees as shown in Fig. 4. The *f*_*i*_ field is mostly positive which shows that positive correlations between anomaly time series predominate. The few locations of negative *f*_*i*_ differ in position between datasets. Spatial contrasts between *f*_*i*_ values are least pronounced for TRMM and most for CCLM.

Although SRn and SRp links encode fundamentally different statistical relationships, the degree fields of the respective networks roughly agree in exhibiting and locating three distinct regions of enhanced degree indicated by colored boxes in Fig. 4(i). All models reproduce this general pattern. As to the climatological interpretation of the SRp network we notice that the regions of large degree are adjacent to the zones of most abundant seasonal rainfall (cf. Fig. 2), while over those zones themselves (Amazon basin, ITCZ, SACZ) degrees are low, just like over the very dry southern hemisphere Pacific coast. This is explained as follows. Precipitation time series in very dry regions are mostly constant and can therefore not be correlated to other more rainy, less constant time series. The abundant rainfall in the convergence zones is associated with frequent localized convective rain events [59], hence the low correlation levels here. In contrast, wet and dry periods alternate in the intermediately wet regions adjacent to the convergence zones, which causes large intraregional correlations there. We show below that the SRp network links are indeed purely intraregional. There we also give a climatological interpretation of the SRn network. For a detailed climatological interpretation of the ESp network we refer the reader to [64].

**Fig 5:**
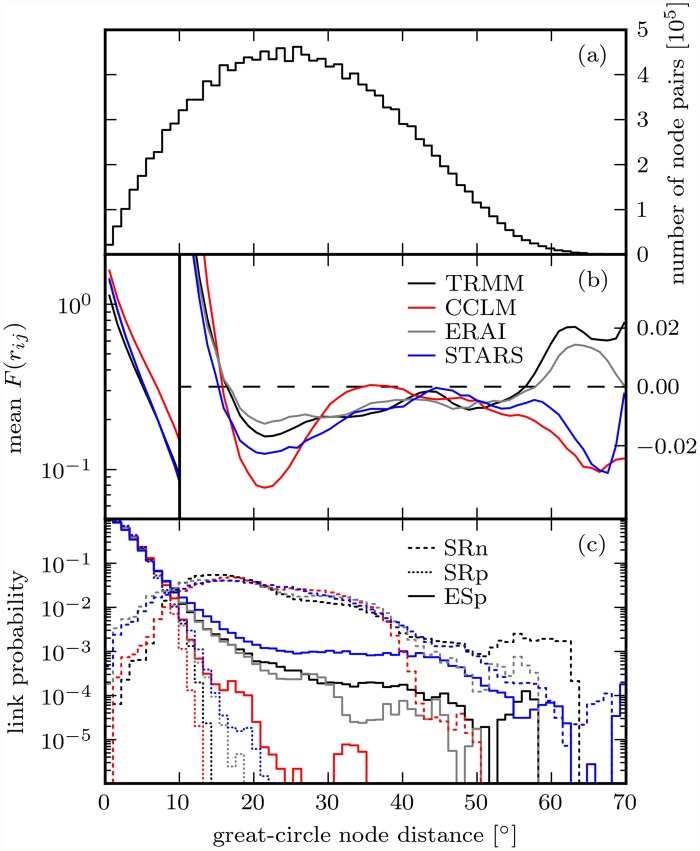
Dependence of rank correlations (b) and simple graph link probabilities (c) on geographical node distance for TRMM, CCLM, ERA and STARS networks, based on binned node distances and their absolute number distribution (a). The link probabilities in (c) are the conditional probabilities of finding two nodes linked given their distance. A great-circle distance of 10° corresponds to a geographical distance of approximately 1113km.

We proceed by analyzing the dependence of mean correlations and simple graph link probabilities on the geographical distance of nodes (Fig. 5). While it is known that correlations between precipitation time series decay with distance [66] and a similar behavior has been found for synchronizations of extreme precipitation events [27], details of the SR and ES decays have not yet been compared and we do not know at which distance to expect the anticorrelations represented by the SRn network.

Fig. 5(b) shows an approximately exponential decay of *F*(*r*_*ij*_) values with node distance for short distances, followed by predominating anticorrelations for intermediate distances from about 15° to 55°. The distribution of *F*(*r*_*ij*_) values becomes bimodal at distances beyond 60°, with positive and negative modes representing correlations and anticorrelations between anomaly time series in the diagonally opposite corners of the domain; deviations of mean *F*(*r*_*ij*_) values from zero at those distances are therefore not statistically significant. While all models reproduce these characteristics, STARS and particularly CCLM overestimate the absolute values of both the most positive and the most negative correlations; ERAI follows the TRMM observations more closely. We find corresponding deviations in the rank correlation thresholds defining the SR simple graphs: The 2nd and 98th percentiles of SR are -0.16/-0.24/-0.18/-0.23 and 0.43/0.58/0.48/0.47 for TRMM/CCLM/ERAI/STARS, respectively.

For the three simple graphs, the link probability as a function of geographical node distance is shown in Fig. 5(c); the respective absolute number distribution of node distances is depicted in Fig. 5(a). Most of the 2% strongest anticorrelations turn out to connect anomaly time series 10° to 40° away from each other. SRp and ESp link probabilities decay differently for distances beyond about 10°. In line with *F*(*r*_*ij*_) values, SRp link probabilities go to zero around 15°, while some nodes much farther apart are linked in the ESp graph. Distinctly positive general rainfall anomaly correlations are hence confined to be short ranged, while some of the 2% strongest extreme rainfall event synchronizations over South America are found between locations thousands of kilometers apart. Across graph types, CCLM produces too many short- and too few long-range links, which means that the model underestimates the strength of teleconnections relative to local anomaly correlations and event synchronizations. ERAI and STARS show less coherent deviations from TRMM with the reanalysis following the observations most closely, overall.

**Fig 6:**
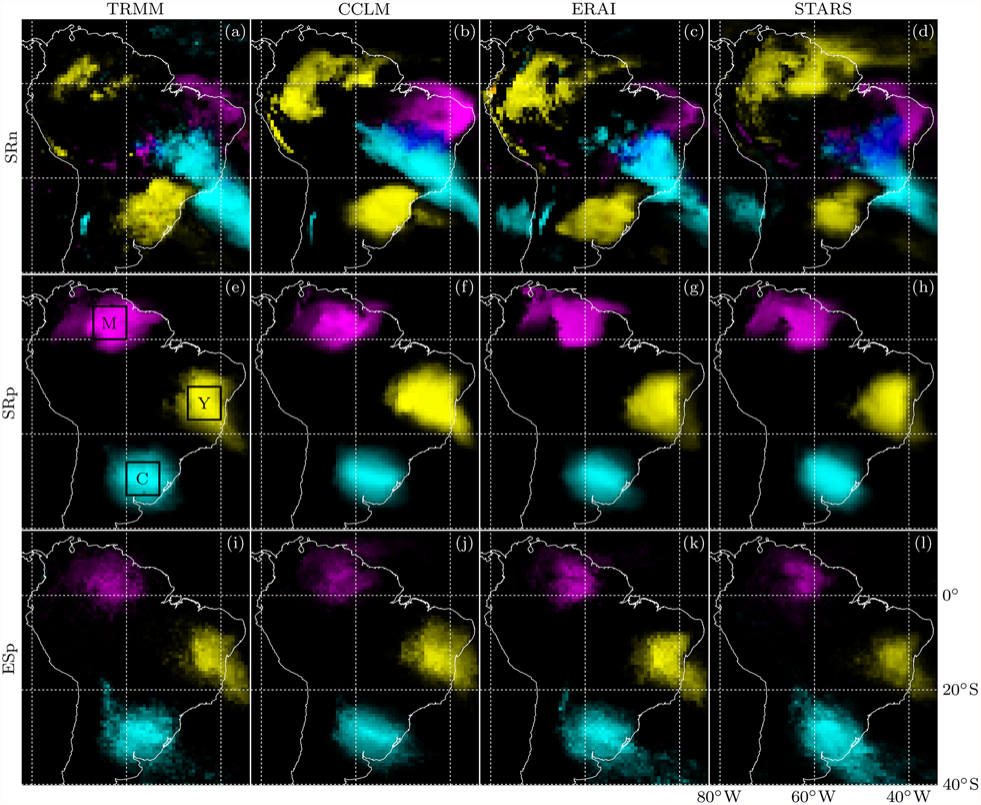
Connectivities to the regions marked by colored boxes in Fig. 4(i) for the TRMM, CCLM, ERAI and STARS (from left to right) SRn, SRp and ESp (from top to bottom) graphs. For each node *i*, the connectivity to the three regions [marked again by black boxes in (e)] is transformed to an RGB color with additive color mixing. We use cyan, magenta and yellow for links to C, M and Y, respectively, with color intensities proportional to the number of links between *i* and the respective region. In formulas, if *i* is connected to *n*_*C*_, *n*_*M*_ and *n*_*Y*_ nodes in boxes C, M and Y, respectively, we calculate an 24-bit RGB color code of (255 *n*_*C*_/100, 255 *n*_*M*_/100, 255 *n*_*Y*_/100) as all boxes contain exactly 100 nodes, and apply an additional hue shift by 180° [67]. Note that, since the hypothetical case of a node connected to all C, M and Y nodes does not occur, white has a purely decorative meaning.

To shed some more light on the network topologies, Fig. 6 depicts RGB color coded connectivities to the three regions C, M, Y defined by colored boxes in Fig. 4(i). In the TRMM SRn graph, we find nodes south of Y connected to C and nodes in and northeast of C connected to Y. Moreover, we find nodes north of Y connected to M and nodes west of M connected to Y. Rainfall dipole patterns underlying these regional connectivities have been studied in the climatological literature. The M-Y anticorrelation has been associated with active and break phases in the South American monsoon system [68]. The C-Y one corresponds to the well-studied SACZ seesaw pattern, which is caused by middle-latitude frontal systems propagating into the tropics [69, 70]. In contrast to SRn, the SRp and ESp graphs are dominated by short-range links and no interregional connections are found. The models reproduce the general patterns of connectivity to the regions C, M, Y for every graph type, yet with reduced accuracy for SRn compared to SRp and ESp. In particular, anticorrelations between C and the maritime SACZ are underestimated by every model (cf. Fig. 4), and CCLM overestimates the strength of the M-Y anticorrelation.

### Network differences

We now study deviations of modeled from observed precipitation networks as quantified by the difference measures introduced above. Yet before going local we take a look at global network differences to analyze variations in climate model performances with network type and to connect to the Feldhoff et al. [8] results.

#### Global network differences

**Fig 7:**
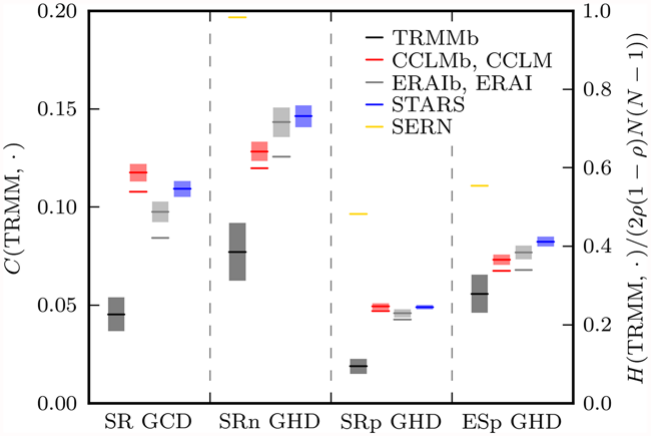
Global correlation and Hamming distances of modeled to TRMM precipitation networks for the four different network types SR, SRn, SRp, ESp (from left to right). The left and right scales apply to the GCDs and GHDs, respectively. The latter have been renormalized by the expectation value of the GHD to an Erdős-Rényi random network with the same link density [8, 29, 71]. For ensemble networks (TRMMb, CCLMb, ERAIb, STARS, SERN, 200 realizations each), the range of ±1 standard deviation around the ensemble mean is shaded. Lines without uncertainty shading represent single realization networks (CCLM, ERAI). The uncertainties of *H*(TRMM, SERN) are invisibly small.

Global correlation and Hamming distances of modeled to TRMM precipitation networks are depicted in Fig. 7. Like Feldhoff et al. [8], we relate climate model performances to those of random network models which preserve certain properties of the respective TRMM network. The GHDs in Fig. 7 have been renormalized by 2*ρ*(1 − *ρ*)*N*(*N*−1), the expected GHD to an Erdős-Rényi random network with the same link density [8, 29, 71]. This type of random graph represents the performance of the worst possible climate model, i.e. one that simulates graphs that have nothing in common with the TRMM graph except the link density.

As a second reference we employ the Spatially Embedded Random Network (SERN) model to account for spatial embedding effects on the network topology [38, 72]. Graphs generated by this model have the same distribution of geographical link lengths as the respective TRMM graph. We have seen that the climate models basically reproduce those distributions with their network-type dependent character [cf. Fig. 5(c)]. The SERN model performance quantifies what could be expected from a correct such reproduction alone; the smaller the GHD between SERNs and the respective TRMM network, the more topological information on the latter is contained in its link length distribution. We generate an ensemble of 200 SERNs.

When comparing CCLM and ERAI performances with the average STARS performance, one should take into account a disadvantage the statistical model has ab initio. Its resampling algorithm does not preserve the time order of its input data, which implies that, even if STARS was fed with data from the evaluation period, its output would only have the true chronology with negligibly small probability. In contrast, CCLM in its present application and ERAI are aimed at reproducing the actual weather history of the evaluation period. Therefore, while some of the differences between TRMM and STARS networks are due to climate variability represented by the ensemble of climate realizations generated with the statistical model, deviations of this origin are precluded between the TRMM, CCLM and ERAI networks.

To estimate the fraction of network differences that arises from interannual climate variability, we introduce ensembles of networks from resampled TRMM, CCLM and ERAI precipitation time series. We resample according to the bootstrapping method described by Feldhoff et al. [8], i.e. seasonal blocks of data are drawn at random with replacement and concatenated such that the lengths of bootstrapped and original time series are equal. To ensure spatial coherence, every draw is done synchronously at all locations. We construct ensembles of each 200 TRMMb, CCLMb and ERAIb networks from the respective bootstrapped space-time series. This approach is inspired by STARS’ resampling algorithm which, in its first step, does just such a bootstrapping with annual blocks of data [8, Fig. 1].

Fig. 7 reveals that performance differences between CCLM and CCLMb or ERAI and ERAIb are minor which shows that CCLM and ERAI global network differences to TRMM mainly reflect model deficiencies. Nevertheless, in the following we will compare STARS network errors to those of CCLMb and ERAIb only and thereby facilitate a fairer model intercomparison.

From the bootstrap network ensemble spread of GCDs and GHDs we can also learn something about the interannual variability present in each dataset. Irrespective of their type, networks from bootstrapped observational data feature spreads about twice as large as those from bootstrapped reanalysis data and even less variability is present in the CCLMb and STARS ensemble data. The SRp graph exhibits the smallest interannual variability of all simple graphs—a persistence that is arguably due to the predominance of short-range links in this network [73].

Coming back to the SERN model, we observe that its performance varies considerably between graph types. These variations are associated with differences between the respective geographical link length distributions [cf. Fig. 5(c)]. In case of the SRp graph, the distribution is highly informative about the network topology. It allows to infer that only short-range links exist in the network and that geographical neighbors are most likely also topological neighbors. Aside from the existence of several long-range links, this also holds true for the ESp graph, hence the superiority of the SERN model over the Erdős-Rényi model for these two graph types. Analogously, the contrastingly poor SERN model performance for the SRn graph is due to the flatness of the respective link length distribution.

**Fig 8:**
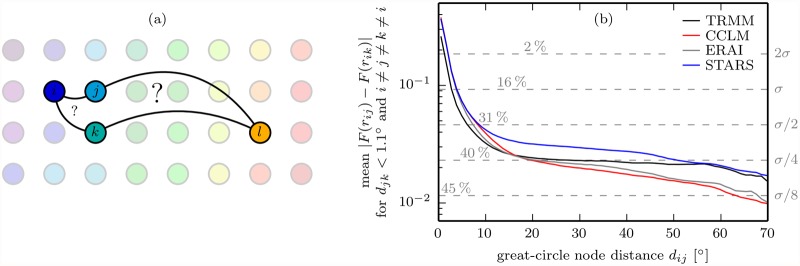
Why in our networks long-range links are statistically more susceptible to misplacement than short-range links. (a) Schematic of a simple graph where the color of a node represents the time series behind it with similarity between colors corresponding to similarity between time series. Note that just like the networks studied, this schematic graph features roughly constant nearest geographical neighbor distances and decreasing node similarity with increasing geographical node distance. (b) Mean absolute differences between Fisher transformed correlation coefficients *r*_*ij*_, *r*_*ik*_ for geographically close nodes *j*, *k* versus *d*_*ij*_, the geographical distance between nodes *i*, *j*. Since the estimation errors of the Fisher transformed rank correlations are value-independent [41, 42], plot (b) shows that with increasing *d*_*ij*_, correlation differences *r*_*ij*_ − *r*_*ik*_ for geographically close nodes *j*, *k* become less robust with respect to the estimation errors of *r*_*ij*_, *r*_*ik*_. As *r*_*ij*_ − *r*_*ik*_ becomes less robust, so does the relative ranking of *r*_*ij*_, *r*_*ik*_ and, hence, the thresholding of *r*_*ij*_, *r*_*ik*_ to *a*_*ij*_, *a*_*ik*_ [Eq. (21)]. Therefore, in the SRn and SRp graphs, the misplacement of a long-range link [*j*-*l* versus *k*-*l* in (a)] is more likely than the misplacement of a short-range link [*i*-*j* versus *i*-*k* in (a)]. To give an idea of link misplacement likelihoods, probabilities of false relative rankings of *r*_*ij*_, *r*_*ik*_ at different *σ* levels are marked in (b). The *σ* levels were calculated using a BART estimator for the effective sample size *M* ^′^ < *M* to account for autocorrelations in the anomaly time series [75].

Conspicuously, SERN and climate model performances vary quite similarly between graph types. Since SERNs and climate model networks have nothing in common but their link length distribution [cf. Fig. 5(c)], the similarity must have something to do with the latter. Yet the explanation of the performance differences between graph types cannot be the same for the random network model and the climate models since CCLM, ERAI and STARS place links in a manner that is clearly not random (cf. Fig. 6). We think that the differences in climate model performances between graph types can be explained by mean geographical link length differences between graph types in connection with the conjecture that in our networks long-range links are statistically more susceptible to misplacement than short-range links. In Fig. 8(b) we give a numerical validation of this conjecture for our SR networks; an analogous validation for the ES network is unfeasible at this point as no variance-stabilizing transformation is known for the ES. A heuristic motivation of the conjecture is possible with the help of an analog from psychology, regarding the network nodes in Fig. 8(a) as grown-up humans, for which, owing to the developmental process of perceptual narrowing [74], it is easier to discriminate between familiar than between unfamiliar types of perceptual stimuli: Because nodes *j*, *k* are more similar to the close node *i* than to the distant node *l*, it is easier for *i* than for *l* to distinguish between *j*, *k*. Thus, in terms of links, *j*-*l* is more likely to be confused with *k*-*l* than *i*-*j* with *i*-*k*. The connection between node similarity and link dissimilarity that is implicit to this analog is established via the statistical dependence measures SR and ES and constitutes the core of the conjecture.

In simple terms, the SRn graph is harder to reproduce than the other simple graphs because its links are longer. We propose to account for this purely geometric-statistical effect by relating climate model performances to the respective SERN model performance which nicely quantifies the effect. And just as *H*(TRMM, SERN) represents a worst case performance scenario, the GCDs and GHDs between TRMM and TRMMb networks constitute optimal performance limits for CCLMb, ERAIb and STARS. In the sense of [76], relating climate model performances to these benchmarks yields a more informative measure of the actual climate model skills when comparing performances for different network types. We thus define a model skill score taking both benchmarks into account by

$$\begin{array}{c} S\left(A;R,O,P\right)=\frac{H(R,P)-H(R,A)}{H(R,P)-H(R,O)} \end{array}$$ (23)

for model *A* with reference *R* = TRMM, optimum *O* = TRMMb and pessimum *P* = SERN. This yields average (over CCLMb, ERAIb and STARS) model skill scores of 0.480(8)/0.624(4)/0.606(11) for SRn/SRp/ESp, respectively. Like Fig. 6, this suggests that the SRn graph is hardest to model, even after taking the link length effect into account. As per Welch’s *t*-test [77], the average SRp and ESp skill scores are not significantly different at the 5% *α* level. Compared to the untransformed GHDs, the skill scores display considerably increased similarity between network types which means that large parts of the performance differences between network types can be attributed to type-specific network uncertainties due to climate variability and spatial embedding.

A model performance intercomparison for the individual network types is permitted as all differences between ensemble mean GCD or GHD values are significant at the 5% *α* level according to Welch’s *t*-test. We find that STARS performs worse than ERAI for every network type considered. The best models are ERAI for the edge-weighted SR network and the SRp graph, and CCLM for the SRn and ESp graphs. The poor SR performance of CCLM is in line with its abovementioned overestimation of absolute correlation values [cf. Fig. 5(b)].

#### Local network differences

**Fig 9:**
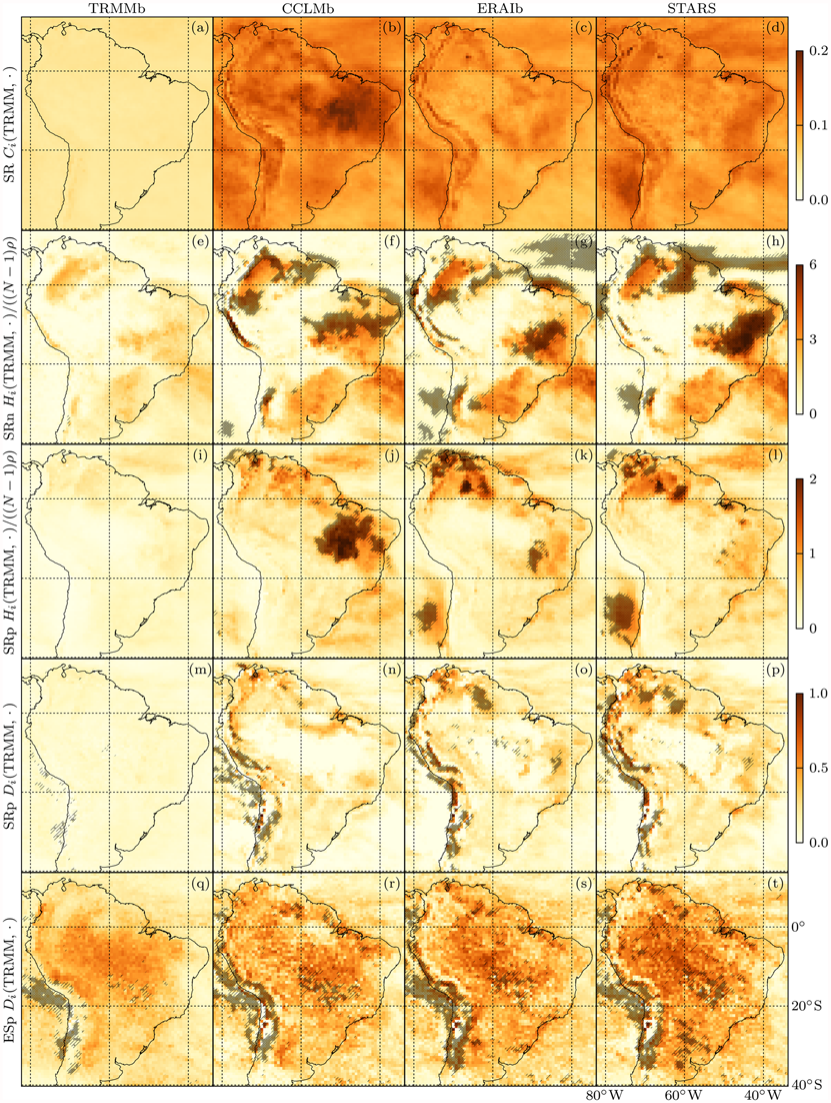
Ensemble mean local differences of TRMMb, CCLMb, ERAIb and STARS (from left to right) to TRMM precipitation networks. SR (a-d) network LCDs [Eq. (11)], SRn (e-h) and SRp (i-l) graph LHDs [Eq. (4)], SRp (m-p) and ESp (q-t) graph DNDs [Eq. (10)]. Note that the LHDs have been renormalized by (*N*−1)*ρ*, analogously to the degrees (Fig. 4). The color scale next to (p) is also applicable to (q-t). Hatching in (e-l) puts LHDs in relation to *H*_*i*_(TRMM, SERN) of the respective graph type; same for DNDs in (m-t); light hatching indicates insignificantly different values at the 5% *α* level according to Welch’s *t*-test [77]; heavy (no) hatching indicates a significantly better (worse) local performance by the random network model.

We now come to the application of the new local network difference measures. LCDs, LHDs and DNDs between TRMM and model precipitation networks are depicted in Fig. 9. We do not show the LHDs between ESp graphs because they are dominated by the degree dependence which motivated the introduction of the DND, nor the DNDs between SRn graphs since they are undefined in many locations due to a multitude of isolated nodes in these graphs.

Starting with the LCD, we find virtually no spatial variability in *C*_*i*_(TRMM, TRMMb), which is due to the use of the Fisher transformation in the LCD definition [Eq. (11)] if we consider differences between TRMM and TRMMb correlations to quantify estimation errors. The contrasting spatial dependence of climate model LCDs hints at model deficiencies with spatially inhomogeneous consequences. Higher correlation differences common to all models can be observed along the Andes, in northeastern Brazil and over the Pacific south of 20°S while lower values occur over the Atlantic south of the SACZ. We observe LCD contrasts along the coasts and find a larger LCD spread across models over land than over sea. The largest correlation distances to TRMM are produced by CCLM over central Brazil.

The LHDs of SRn and SRp simple graphs are clearly degree-dependent (cf. Fig. 4). We see that high LCDs may coincide with high LHDs as for instance over the Pacific off the Chilean coast for STARS or over central and northeastern Brazil for CCLM. Yet where correlations are not strong enough to be represented by links in the simple graphs, high LCDs may come along with low LHDs as can be observed along the Andes.

Hatching in Fig. 9(e-t) visualizes the respective relative local SERN model performances. Light hatching indicates insignificantly different local network differences at the 5% *α* level according to Welch’s *t*-test [77] while heavy (no) hatching indicates a significantly better (worse) local performance by the random network model. In line with GHD results (cf. Fig. 7), the climate models perform better in most locations. Yet there are areas where mere knowledge of the observed all-domain link length distribution allows for a better neighborhood prediction than the use of a climate model. With the LHD, these areas differ considerably between models.

A more coherent picture only emerges with the DND, shown for SRp and ESp graphs in Figs. 9(m-p) and 9(q-t), respectively. We observe larger DNDs over land than over sea and find particularly high values in the Guyana Highlands and along the Andes. DNDs between TRMM and TRMMb are much larger for the ESp than for the SRp graph. Climatic uncertainties of extreme event synchronizations are most pronounced in the Amazon basin and along the eastern slopes of the Andes, which is consistent with the outstanding noisiness of TRMM’s ESp degree field in those areas [cf. Fig. 4(m)]. In line with the respective GHDs (Fig. 7), the ESp DNDs to TRMM increase slightly from TRMMb to CCLMb to ERAIb to STARS while their spatial patterns do not vary much across datasets. This shows that most of the differences between TRMM and climate model ESp graphs can be explained with interannual climate variability.

In this context, note that, generally, the local network difference fields based on the original CCLM and ERAI time series do not differ much from the respective bootstrap ensemble mean fields. The latter are smoother and feature slightly larger values but apart from that show the same spatial patterns. Hence, where climate model bootstrap network differences to TRMM clearly exceed the corresponding difference between TRMM and TRMMb, network imperfections cannot be explained by climatic uncertainty but must be due to model deficiencies.

## Discussion

With the present study we advance the complex network approach to climate model evaluation recently put forward by Feldhoff et al. [8]. We do so in three ways, going from the general to the particular. Firstly, we expose the wider scope of application of the approach outlining how it can be used to evaluate models of real-world multidimensional dynamical systems of any background; secondly, we define local versions of the global network difference measures introduced by Feldhoff et al. [8] to facilitate spatially explicit network comparisons in the climate model evaluation context; and thirdly, we apply both local and global network difference measures in a sample climate model evaluation where we highlight intricacies of the approach related to comparisons of model performances across network types.

The first local network difference measure we define is a local version of the global Hamming distance (GHD) between simple graphs. We demonstrate that the local Hamming distance (LHD) comes with a disadvantageous degree dependence, which we propose to overcome using a suitable statistical null model, relating the actual LHD value to the null model probability distribution of possible LHD values given the degrees of the respective node. This way a second local network difference measure is defined which we call the degree-conditional neighborhood dissimilarity (DND).

Our ansatz to render the LHD degree-independent via a statistical null model goes beyond existing ad hoc renormalization methods used by the vertex similarity community to solve the equivalent problem occurring when comparing different nodes of the same network [21]. As our ansatz has the virtue of an explicit probabilistic motivation and is easily transferred to the equivalent vertex similarity problem, we propose it to supersede the ad hoc approaches common in that field.

By definition, the relationship between network LHDs and DNDs is similar to the one between absolute and relative precipitation biases. Large LHDs can coincide with small DNDs and vice versa. The two network difference measures should therefore always be considered conjointly to prevent misinterpretations. Notably, while zero LHDs imply zero DNDs, the converse is not true. Rather, a vanishing DND indicates the greatest possible neighborhood overlap.

We also examine the case of edge-weighted graphs represented by matrices of statistical relationship coefficients. It is argued that difference measures for such graphs should account for estimation uncertainties inherent to the coefficients. For this purpose we propose to employ variance-stabilizing transformations like the Fisher transformation in the case of correlations. Using the latter we define the local and the global correlation distance (LCD, GCD) between correlation matrices in formal analogy to the LHD and the GHD between simple graphs.

Generalizations of our local network difference measures to node-weighted graphs are presented as well. Computing LHDs and DNDs between directed graphs is straightforwardly done by separating ingoing and outgoing links, i.e. by defining $H_{i}^{\pm }$ and in turn $D_{i}^{\pm }$ via $k_{i}^{A\pm },k_{i}^{B\pm }$. The new difference measures are therefore highly versatile and can be used to compare networks of any functional or structural background as long as these have a common set of nodes. They might be useful additional tools for investigations of network evolution, such as in [73, 78], or for studying the impact of disorders and disasters on network topologies, similar to [79–82].

In a sample climate model evaluation we apply the network difference measures to comparatively analyze statistical and dynamical regional climate simulations of the South American monsoon system. We focus on climate networks constructed from DJF precipitation space-time series and use satellite data provided by the Tropical Rainfall Measuring Mission (TRMM) 3B42 V7 as a reference. We also include the ERA-Interim (ERAI) reanalysis data in the evaluation since they where used to drive the simulations of both the statistical model STARS and the dynamical model CCLM.

Different types of networks are constructed to capture distinct aspects of the spatiotemporal precipitation dynamics. Based on spatial rank correlations between anomaly time series we define an edge-weighted graph called SR and two simple graphs with links representing the 2% most positive and the 2% most negative correlations called SRp and SRn, respectively. Another simple graph called ESp is based on the 2% strongest spatial synchronizations between extreme event time series. We show that the ESp and SRp graphs are dominated by short-range links while teleconnections prevail in the SRn graph. Visualizing these teleconnections using an RGB color model we reveal that they represent the two major rainfall dipoles of the South American monsoon system.

Global distances between TRMM and ERAI/CCLM/STARS simple graphs vary strongly across network types, which allows us to uncover how climate variability and spatial embedding effects pose limits to the fidelity of network reproduction. It is shown that as geographical link lengths increase, so does the likelihood of their misplacement, which explains why GHDs are much larger for the SRn than for the SRp and ESp graphs. We employ spatially embedded random networks (SERNs) to model this effect. The fraction of network differences due to interannual climate variability is quantified using a bootstrap approach. In agreement with the fickle nature of extreme events, we find the ESp graph most affected by this source of uncertainty. Based on SERN and bootstrap network differences to TRMM, a model skill score is defined that accounts for the named uncertainties. According to this score, the SRp and ESp graphs are reproduced with similar and higher fidelity than the SRn graph. This result makes sense inasmuch as more than the former graphs, the latter contains nontrivial information about the system dynamics.

Comparing performances across reanalysis and climate models, we find that CCLM beats ERAI at two out of four network types, while STARS performs worse than ERAI in all cases. As both CCLM and STARS were driven by ERAI data, this suggests that dynamical downscaling may retain value at the resolution of the driving data while statistical resampling can only impair data quality. Interestingly, for the SRp graph, our evaluation against the independent TRMM data yields a relative performance ranking of CCLM versus STARS that is opposite to the one obtained in an evaluation against the driving reanalysis data [8].

From the ensemble spread of bootstrap network distances to TRMM we infer that ERAI and even more so CCLM and STARS underestimate the interannual variability of the precipitation dynamics encoded in our networks. As for the reanalysis, this finding complements documented deficiencies in reproducing the variability of spatiotemporal totals of tropical rainfall over land [83, 84]. The loss of large-scale variability in the CCLM simulations might have been prevented by spectral nudging [85, 86]. In case of STARS we conclude that its resampling algorithm tends to reduce the variability present in its input data. We suspect deficiencies in cloud physics parameterizations of contributing substantially to the variability underestimations by ERAI and CCLM as simulated precipitation characteristics over monsoon season South America are highly sensitive to modifications of these parameterizations [55] while they are known to lack flexibility in reacting to variations in the large-scale environment [83, 87]. Another factor are model parameters that in reality vary from year to year but are represented by climatological values in ERAI [46, 88] and CCLM [89, 90], like aerosol concentrations, forest albedos or leaf area indices, which means that the models are unable to account for interannual variabilities in aerosol or land-atmosphere feedbacks due to, e.g. volcanic eruptions, bushfires or droughts.

Finally, our new local difference measures are employed to study the spatial distribution of network dissimilarities. In doing so, the DND proves to be most useful to identify commonalities across datasets. Larger DNDs over land than over sea are consistent with land surface-atmosphere interactions being more complex than sea surface-atmosphere interactions, and relatively high DNDs in the Guyana highlands and along the Andes confirm model deficiencies in simulating precipitation over complex terrain [91, 92]. Using the DND we also reveal that for the given observational record length, differences between ESp graphs are dominated by the interannual variability of extreme events, which prevents a reasonable evaluation of their spatial synchronizations at this point.

The situation is different for the correlation networks and we find model-specific spatial patterns in the LHD and LCD fields. The largest SR, SRn and SRp network distances of CCLM to TRMM are found over central and northeastern Brazil. We suppose these biases to be associated with a less severe underestimation of precipitation over this region relative to over the western Amazon basin, and with an erroneous northward displacement of the SACZ [55]. For STARS, the largest correlation network distances to TRMM occur over the Pacific off the Chilean coast. During DJF this region is controlled by a subtropical high that is occasionally disturbed by troughs carried along by the Westerlies of the Southern Hemisphere. Considering that STARS’ simulation domain in fact extents to the southern tip of South America [8], we suppose that the statistical model inadequately resamples the synoptic features that govern the austral summer climate over the southern Pacific Horse Latitudes. A common deficiency of ERAI, CCLM and STARS is an underestimation of the relative strength of the maritime part of the SACZ precipitation seesaw. As both the reanalysis and the dynamical model employ prescribed sea surface temperatures [46, 55], this might be due to an imperfect ocean-atmosphere coupling [93].

## Supporting Information

S1 Data TRMM 3B42 V7 daily precipitation estimates over South America from 1998 to 2011, conservatively interpolated to the ERA-Interim grid. (ZIP)

S2 Data COSMO-CLM 4.25.3 model output of daily precipitation over South America from 1998 to 2011, conservatively interpolated to the ERA-Interim grid. (ZIP)

S3 Data ERA-Interim reanalysis daily precipitation over South America from 1998 to 2011. (ZIP)

S4 Data ERA-Interim reanalysis daily precipitation over South America from 1979 to 1995 and 200 day-to-day mappings from 1996–2011 to 1979–1995 generated with STARS 2.4. From these data it is possible to reconstruct the ensemble of STARS precipitation space-time series used in this study.
(ZIP)

## References

[1] Newman M (2003) The Structure and Function of Complex Networks. SIAM Rev 45: 167–256. 10.1137/S003614450342480

[2] Boccaletti S, Latora V, Moreno Y, Chavez M, Hwang DU (2006) Complex networks: Structure and dynamics. Phys Rep 424: 175–308. 10.1016/j.physrep.2005.10.009

[3] Tsonis AA, Swanson KL, Roebber PJ (2006) What Do Networks Have to Do with Climate? Bull Am Meteorol Soc 87: 585–595. 10.1175/BAMS-87-5-585

[4] Donges JF, Zou Y, Marwan N, Kurths J (2009) The backbone of the climate network. Europhys Lett 87: 48007 10.1209/0295-5075/87/48007

[5] Ludescher J, Gozolchiani A, Bogachev MI, Bunde A, Havlin S, et al (2013) Improved El Niño forecasting by cooperativity detection. Proc Natl Acad Sci 110: 11742–11745. 10.1073/pnas.1309353110 23818627 PMC3718177

[6] Bullmore E, Sporns O (2009) Complex brain networks: graph theoretical analysis of structural and functional systems. Nat Rev Neurosci 10: 186–198. 10.1038/nrn2575 19190637

[7] Bressler SL, Menon V (2010) Large-scale brain networks in cognition: emerging methods and principles. TiCS 14: 277–290. 10.1016/j.tics.2010.04.004 20493761

[8] Feldhoff JH, Lange S, Volkholz J, Donges JF, Kurths J, et al (2014) Complex networks for climate model evaluation with application to statistical versus dynamical modeling of South American climate. Clim Dyn (in press).

[9] Erbach-Schoenberg Ez, Bullock S, Brailsford S (2014) A Model of Spatially Constrained Social Network Dynamics. Soc Sci Comput Rev 32: 373–392. 10.1177/0894439313511934

[10] Lux T, Marchesi M (1999) Scaling and criticality in a stochastic multi-agent model of a financial market. Nature 397: 498–500. 10.1038/17290

[11] Zhou C, Zemanová L, Zamora G, Hilgetag CC, Kurths J (2006) Hierarchical Organization Unveiled by Functional Connectivity in Complex Brain Networks. Phys Rev Lett 97: 238103 10.1103/PhysRevLett.97.238103 17280251

[12] Vértes PE, Alexander-Bloch AF, Gogtay N, Giedd JN, Rapoport JL, et al (2012) Simple models of human brain functional networks. Proc Natl Acad Sci 109: 5868–5873. 10.1073/pnas.1111738109 22467830 PMC3326510

[13] de Jong H (2002) Modeling and Simulation of Genetic Regulatory Systems: A Literature Review. J Comput Biol 9: 67–103. 10.1089/10665270252833208 11911796

[14] Ho JWK, Charleston MA (2011) Network modelling of gene regulation. Biophys Rev 3: 1–13. 10.1007/s12551-010-0041-4 28510232 PMC5418390

[15] Fountalis I, Bracco A, Dovrolis C (2013) Spatio-temporal network analysis for studying climate patterns. Clim Dyn 42: 879–899. 10.1007/s00382-013-1729-5

[16] Steinhaeuser K, Tsonis AA (2013) A climate model intercomparison at the dynamics level. Clim Dyn 42: 1665–1670. 10.1007/s00382-013-1761-5

[17] Andrade RFS, Miranda JGV, Pinho STR, ao TPL (2008) Measuring distances between complex networks. Phys Let A 372: 5265–5269. 10.1016/j.physleta.2008.06.044

[18] Papadimitriou P, Dasdan A, Garcia-Molina H (2010) Web graph similarity for anomaly detection. JISA 1: 19–30.

[19] Faloutsos C, Vogelstein JT, Koutra D (2013) DeltaCon: A Principled Massive-Graph Similarity Function. In: Proceedings of the 2013 SIAM International Conference on Data Mining. pp. 162–170.

[20] Lorrain F, White HC (1971) Structural equivalence of individuals in social networks. J Math Sociol 1: 49–80. 10.1080/0022250X.1971.9989788

[21] Leicht EA, Holme P, Newman MEJ (2006) Vertex similarity in networks. Phys Rev E 73: 026120 10.1103/PhysRevE.73.026120 16605411

[22] Blondel V, Gajardo A, Heymans M, Senellart P, Van Dooren P (2004) A Measure of Similarity between Graph Vertices: Applications to Synonym Extraction and Web Searching. SIAM Rev 46: 647–666. 10.1137/S0036144502415960

[23] Zager LA, Verghese GC (2008) Graph similarity scoring and matching. Appl Math Lett 21: 86–94. 10.1016/j.aml.2007.01.006

[24] Tsonis AA, Roebber PJ (2004) The architecture of the climate network. Physica A 333: 497–504. 10.1016/j.physa.2003.10.045

[25] Achard S, Salvador R, Whitcher B, Suckling J, Bullmore E (2006) A Resilient, Low-Frequency, Small-World Human Brain Functional Network with Highly Connected Association Cortical Hubs. J Neurosci 26: 63–72. 10.1523/JNEUROSCI.3874-05.2006 16399673 PMC6674299

[26] Stam CJ, van Dijk BW (2002) Synchronization likelihood: an unbiased measure of generalized synchronization in multivariate data sets. Physica D 163: 236–251. 10.1016/S0167-2789(01)00386-4

[27] Malik N, Bookhagen B, Marwan N, Kurths J (2012) Analysis of spatial and temporal extreme monsoonal rainfall over South Asia using complex networks. Clim Dyn 39: 971–987. 10.1007/s00382-011-1156-4

[28] Hartman D, Hlinka J, Paluš M, Mantini D, Corbetta M (2011) The role of nonlinearity in computing graph-theoretical properties of resting-state functional magnetic resonance imaging brain networks. Chaos 21: 013119 10.1063/1.3553181 21456833 PMC4108645

[29] Donges JF, Zou Y, Marwan N, Kurths J (2009) Complex networks in climate dynamics. Eur Phys J ST 174: 157–179. 10.1140/epjst/e2009-01098-2

[30] Simpson SL, Bowman FD, Laurienti PJ (2013) Analyzing complex functional brain networks: Fusing statistics and network science to understand the brain. Statist Surv 7: 1–36. 10.1214/13-SS103 PMC4189131 25309643

[31] Hamming RW (1950) Error Detecting and Error Correcting Codes. Bell Syst Tech J 29: 147–160. 10.1002/j.1538-7305.1950.tb00463.x

[32] Jaccard P (1901) Étude comparative de la distribution florale dans une portion des Alpes et du Jura. Bull Soc Vaud Sci Nat 37: 547–579.

[33] Salton G (1989) Automatic Text Processing: The Transformation, Analysis, and Retrieval of Information by Computer. Addison-Wesley.

[34] Ravasz E, Somera AL, Mongru DA, Oltvai ZN, Barabási AL (2002) Hierarchical Organization of Modularity in Metabolic Networks. Science 297: 1551–1555. 10.1126/science.1073374 12202830

[35] Graham RL, Knuth DE, Patashnik O (1989) Concrete mathematics: a foundation for computer science. Addison-Wesley.

[36] Petkovšek M, Wilf HS, Zeilberger D (1996) A = B. AK Peters Ltd.

[37] Berkopec A (2007) HyperQuick algorithm for discrete hypergeometric distribution. J Discret Algorithms 5: 341–347. 10.1016/j.jda.2006.01.001

[38] Rheinwalt A, Marwan N, Kurths J, Werner P, Gerstengarbe FW (2012) Boundary effects in network measures of spatially embedded networks. Europhys Lett 100: 28002 10.1209/0295-5075/100/28002

[39] Fisher RA (1915) Frequency Distribution of the Values of the Correlation Coefficient in Samples from an Indefinitely Large Population. Biometrika 10: 507–521. 10.1093/biomet/10.4.507

[40] Fisher RA (1921) On the “probable error” of a coefficient of correlation deduced from a small sample. Metron 1: 3–32.

[41] Fieller EC, Hartley HO, Pearson ES (1957) Tests for Rank Correlation Coefficients. I. Biometrika 44: 470–481. 10.1093/biomet/44.3-4.470

[42] Fieller EC, Pearson ES (1961) Tests for Rank Correlation Coefficients: II. Biometrika 48: 29–40. 10.1093/biomet/48.1-2.29

[43] Heitzig J, Donges JF, Zou Y, Marwan N, Kurths J (2012) Node-weighted measures for complex networks with spatially embedded, sampled, or differently sized nodes. Eur Phys J B 85: 1–22. 10.1140/epjb/e2011-20678-7

[44] Hörmann W (1994) A universal generator for discrete log-concave distributions. Computing 52: 89–96. 10.1007/BF02243398

[45] Bagnoli M, Bergstrom T (2005) Log-concave probability and its applications. Econ Theory 26: 445–469. 10.1007/s00199-004-0514-4

[46] Dee DP, Uppala SM, Simmons AJ, Berrisford P, Poli P, et al (2011) The ERA-Interim reanalysis: configuration and performance of the data assimilation system. Quart J Roy Meteor Soc 137: 553–597. 10.1002/qj.828

[47] Kalnay E, Kanamitsu M, Kistler R, Collins W, Deaven D, et al (1996) The NCEP/NCAR 40-Year Reanalysis Project. Bull Am Meteorol Soc 77: 437–471. 10.1175/1520-0477(1996)077<0437:TNYRP>2.0.CO;2

[48] Trenberth KE, Stepaniak DP, Hurrell JW, Fiorino M (2001) Quality of Reanalyses in the Tropics. J Clim 14: 1499–1510. 10.1175/1520-0442(2001)014<1499:QORITT>2.0.CO;2

[49] Marshall GJ (2002) Trends in Antarctic Geopotential Height and Temperature: A Comparison between Radiosonde and NCEP-NCAR Reanalysis Data. J Clim 15: 659–674. 10.1175/1520-0442(2002)015<0659:TIAGHA>2.0.CO;2

[50] Rockel B, Will A, Hense A (2008) The Regional Climate Model COSMO-CLM (CCLM). Meteorol Z 17: 347–348. 10.1127/0941-2948/2008/0309

[51] Baldauf M, Seifert A, Förstner J, Majewski D, Raschendorfer M, et al (2011) Operational Convective-Scale Numerical Weather Prediction with the COSMO Model: Description and Sensitivities. Mon Weather Rev 139: 3887–3905. 10.1175/MWR-D-10-05013.1

[52] Werner PC, Gerstengarbe FW (1997) Proposal for the development of climate scenarios. Clim Res 8: 171–182. 10.3354/cr008171

[53] Orlowsky B, Gerstengarbe FW, Werner PC (2008) A resampling scheme for regional climate simulations and its performance compared to a dynamical RCM. Theor Appl Climatol 92: 209–223. 10.1007/s00704-007-0352-y

[54] Giorgi F, Jones C, Asrar GR (2009) Addressing climate information needs at the regional level: the CORDEX framework. WMO Bulletin 58: 175–183.

[55] Lange S, Rockel B, Volkholz J, Bookhagen B (2014) Regional climate model sensitivities to parametrizations of convection and non-precipitating subgrid-scale clouds over South America. Clim Dyn (in press).

[56] Jones PW (1999) First- and Second-Order Conservative Remapping Schemes for Grids in Spherical Coordinates. Mon Weather Rev 127: 2204–2210. 10.1175/1520-0493(1999)127<2204:FASOCR>2.0.CO;2

[57] Huffman GJ, Bolvin DT, Nelkin EJ, Wolff DB, Adler RF, et al (2007) The TRMM Multisatellite Precipitation Analysis (TMPA): Quasi-Global, Multiyear, Combined-Sensor Precipitation Estimates at Fine Scales. J Hydrometeorol 8: 38–55. 10.1175/JHM560.1

[58] Zhou J, Lau KM (1998) Does a Monsoon Climate Exist over South America? J Clim 11: 1020–1040. 10.1175/1520-0442(1998)011<1020:DAMCEO>2.0.CO;2

[59] Vera C, Higgins W, Amador J, Ambrizzi T, Garreaud R, et al (2006) Toward a Unified View of the American Monsoon Systems. J Clim 19: 4977–5000. 10.1175/JCLI3896.1

[60] Marengo JA, Liebmann B, Grimm AM, Misra V, Silva Dias PL, et al (2012) Recent developments on the South American monsoon system. Int J Climatol 32: 1–21. 10.1002/joc.2254

[61] Dai A (2006) Precipitation Characteristics in Eighteen Coupled Climate Models. J Clim 19: 4605–4630. 10.1175/JCLI3884.1

[62] Quiroga RQ, Kreuz T, Grassberger P (2002) Event synchronization: A simple and fast method to measure synchronicity and time delay patterns. Phys Rev E 66: 041904 10.1103/PhysRevE.66.041904 12443232

[63] Spearman C (1904) The Proof and Measurement of Association between Two Things. Am J Psychol 15: 72–101. 10.2307/1412159 3322052

[64] Boers N, Bookhagen B, Marwan N, Kurths J, Marengo J (2013) Complex networks identify spatial patterns of extreme rainfall events of the South American Monsoon System. Geophys Res Lett 40: 4386–4392. 10.1002/grl.50681

[65] Kendall MG (1945) The treatment of ties in ranking problems. Biometrika 33: 239–251. 10.1093/biomet/33.3.239 21006841

[66] Huff FA, Shipp WL (1969) Spatial Correlations of Storm, Monthly and Seasonal Precipitation. J Appl Meteorol 8: 542–550. 10.1175/1520-0450(1969)008<0542:SCOSMA>2.0.CO;2

[67] Agoston MK (2005) Computer Graphics and Geometric Modelling, Springer, volume 1 pp. 301–304.

[68] Jones C, Carvalho LMV (2002) Active and Break Phases in the South American Monsoon System. J Clim 15: 905–914. 10.1175/1520-0442(2002)015<0905:AABPIT>2.0.CO;2

[69] Nogués-Paegle J, Mo KC (1997) Alternating Wet and Dry Conditions over South America during Summer. Mon Weather Rev 125: 279–291. 10.1175/1520-0493(1997)125<0279:AWADCO>2.0.CO;2

[70] Carvalho LMV, Jones C, Liebmann B (2004) The South Atlantic Convergence Zone: Intensity, Form, Persistence, and Relationships with Intraseasonal to Interannual Activity and Extreme Rainfall. J Clim 17: 88–108. 10.1175/1520-0442(2004)017<0088:TSACZI>2.0.CO;2

[71] Erdős P, Rényi A (1959) On Random Graphs I. Publ Math Debrecen 6: 290–297.

[72] Barnett L, Di Paolo E, Bullock S (2007) Spatially embedded random networks. Phys Rev E 76: 056115 10.1103/PhysRevE.76.056115 18233726

[73] Radebach A, Donner RV, Runge J, Donges JF, Kurths J (2013) Disentangling different types of El Niño episodes by evolving climate network analysis. Phys Rev E 88: 052807 10.1103/PhysRevE.88.052807 24329318

[74] Scott LS, Pascalis O, Nelson CA (2007) A Domain-General Theory of the Development of Perceptual Discrimination. Curr Dir Psychol 16: 197–201. 10.1111/j.1467-8721.2007.00503.x PMC2995946 21132090

[75] Thiébaux HJ, Zwiers FW (1984) The Interpretation and Estimation of Effective Sample Size. J Clim Appl Meteorol 23: 800–811. 10.1175/1520-0450(1984)023<0800:TIAEOE>2.0.CO;2

[76] Murphy AH (1993) What Is a Good Forecast? An Essay on the Nature of Goodness in Weather Forecasting. Wea Forecasting 8: 281–293. 10.1175/1520-0434(1993)008<0281:WIAGFA>2.0.CO;2

[77] Welch BL (1947) The generalization of ‘Student’s’ problem when several different population varlances are involved. Biometrika 34: 28–35. 10.1093/biomet/34.1-2.28 20287819

[78] Spoormaker VI, Schröter MS, Gleiser PM, Andrade KC, Dresler M, et al (2010) Development of a Large-Scale Functional Brain Network during Human Non-Rapid Eye Movement Sleep. J Neurosci 30: 11379–11387. 10.1523/JNEUROSCI.2015-10.2010 20739559 PMC6633325

[79] Greicius MD, Flores BH, Menon V, Glover GH, Solvason HB, et al (2007) Resting-State Functional Connectivity in Major Depression: Abnormally Increased Contributions from Subgenual Cingulate Cortex and Thalamus. Biol Psychiatry 62: 429–437. 10.1016/j.biopsych.2006.09.020 17210143 PMC2001244

[80] Lynall ME, Bassett DS, Kerwin R, McKenna PJ, Kitzbichler M, et al (2010) Functional Connectivity and Brain Networks in Schizophrenia. J Neurosci 30: 9477–9487. 10.1523/JNEUROSCI.0333-10.2010 20631176 PMC2914251

[81] Woolley-Meza O, Grady D, Thiemann C, Bagrow JP, Brockmann D (2013) Eyjafjallajökull and 9/11: The Impact of Large-Scale Disasters on Worldwide Mobility. PLoS One 8: e69829 10.1371/journal.pone.0069829 23950904 PMC3737197

[82] Levermann A (2014) Comment: Make supply chains climate-smart. Nature 506: 27–29. 10.1038/506027a 24499903

[83] Bechtold P, Köhler M, Jung T, Doblas-Reyes F, Leutbecher M, et al (2008) Advances in simulating atmospheric variability with the ECMWF model: From synoptic to decadal time-scales. Quart J Roy Meteor Soc 134: 1337–1351. 10.1002/qj.289

[84] Nikulin G, Jones C, Giorgi F, Asrar G, Büchner M, et al (2012) Precipitation Climatology in an Ensemble of CORDEX-Africa Regional Climate Simulations. J Clim 25: 6057–6078. 10.1175/JCLI-D-11-00375.1

[85] Castro CL, Pielke RA Sr, Leoncini G (2005) Dynamical downscaling: Assessment of value retained and added using the Regional Atmospheric Modeling System (RAMS). J Geophys Res 110: D05108.

[86] Rockel B, Castro CL, Pielke RA Sr, von Storch H, Leoncini G (2008) Dynamical downscaling: Assessment of model system dependent retained and added variability for two different regional climate models. J Geophys Res 113: D21107 10.1029/2007JD009461

[87] Bechtold P, Semane N, Lopez P, Chaboureau JP, Beljaars A, et al (2014) Representing Equilibrium and Nonequilibrium Convection in Large-Scale Models. J Atmos Sci 71: 734–753. 10.1175/JAS-D-13-0163.1

[88] Loveland TR, Reed BC, Brown JF, Ohlen DO, Zhu Z, et al (2000) Development of a global land cover characteristics database and IGBP DISCover from 1 km AVHRR data. Int J Remote Sens 21: 1303–1330. 10.1080/014311600210191

[89] Smiatek G, Rockel B, Schättler U (2008) Time invariant data preprocessor for the climate version of the COSMO model (COSMO-CLM). Meteorol Z 17: 395–405. 10.1127/0941-2948/2008/0302

[90] Doms G, Förstner J, Heise E, Herzog HJ, Mironov D, et al. (2011) A Description of the Nonhydrostatic Regional COSMO Model, Part II: Physical Parameterization. Deutscher Wetterdienst. COSMO model documentation website: http://www.cosmo-model.org/content/model/documentation/core/default.htm. Accessed 2015 Jan 9.

[91] Bachner S, Kapala A, Simmer C (2008) Evaluation of daily precipitation characteristics in the CLM and their sensitivity to parameterizations. Meteorologische Zeitschrift 17: 407–419. 10.1127/0941-2948/2008/0300

[92] Ward E, Buytaert W, Peaver L, Wheater H (2011) Evaluation of precipitation products over complex mountainous terrain: A water resources perspective. Adv Water Resour 34: 1222–1231. 10.1016/j.advwatres.2011.05.007

[93] Braconnot P, Hourdin F, Bony S, Dufresne J, Grandpeix J, et al (2007) Impact of different convective cloud schemes on the simulation of the tropical seasonal cycle in a coupled ocean-atmosphere model. Clim Dyn 29: 501–520. 10.1007/s00382-007-0244-y

