# Correction: Local Difference Measures between Complex Networks for Dynamical System Model Evaluation

**DOI:** 10.1371/journal.pone.0129413

**Published:** 2015-06-04

**Authors:** Stefan Lange, Jonathan F. Donges, Jan Volkholz, Jürgen Kurths

There are errors in references 8 and 55. The correct references are:

8. Feldhoff JH, Lange S, Volkholz J, Donges JF, Kurths J, Gerstengarbe FW (2014) Complex networks for climate model evaluation with application to statistical versus dynamical modeling of South American climate. Clim Dyn. doi:10.1007/s00382-014-2182-9

55. Lange S, Rockel B, Volkholz J, Bookhagen B (2014) Regional climate model sensitivities to parametrizations of convection and non-precipitating subgrid-scale clouds over South America. Clim Dyn. doi:10.1007/s00382-014-2199-0

There is an error in the reference in the fourth sentence of the Abstract. The sentence should read: Building on a recent study by Feldhoff et al. [8] we comparatively analyze statistical and dynamical regional climate simulations of the South American monsoon system.
